# Physical Exercise Enhanced Heat Shock Protein 60 Expression and Attenuated Inflammation in the Adipose Tissue of Human Diabetic Obese

**DOI:** 10.3389/fendo.2018.00016

**Published:** 2018-02-06

**Authors:** Abdelkrim Khadir, Sina Kavalakatt, Preethi Cherian, Samia Warsame, Jehad Ahmed Abubaker, Mohammed Dehbi, Ali Tiss

**Affiliations:** ^1^Research Division, Dasman Diabetes Institute, Kuwait City, Kuwait; ^2^Diabetes Research Centre, Qatar Biomedical Research Institute, Hamad Bin Khalifa University, Qatar Foundation, Doha, Qatar

**Keywords:** cellular stress, heat shock response, heat shock protein 60, physical exercise, adipose tissue

## Abstract

Heat shock protein 60 (HSP60) is a key protein in the crosstalk between cellular stress and inflammation. However, the status of HSP60 in diabetes and obesity is unclear. In the present study, we investigated the hypothesis that HSP60 expression levels in the adipose tissue of human obese adults with and without diabetes are different and physical exercise might affect these levels. Subcutaneous adipose tissue (SAT) and blood samples were collected from obese adults with and without diabetes (*n* = 138 and *n* = 92, respectively, at baseline; *n* = 43 for both groups after 3 months of physical exercise). Conventional RT-PCR, immunohistochemistry, immunofluorescence, and ELISA were used to assess the expression and secretion of HSP60. Compared with obese adults without diabetes, HSP60 mRNA and protein levels were decreased in SAT in diabetic obese together with increased inflammatory marker expression and glycemic levels but lower VO_2 Max_. More interestingly, a 3-month physical exercise differentially affected HSP60 expression and the heat shock response but attenuated inflammation in both groups, as reflected by decreased endogenous levels of IL-6 and TNF-α. Indeed, HSP60 expression levels in SAT were significantly increased by exercise in the diabetes group, whereas they were decreased in the non-diabetes group. These results were further confirmed using immunofluorescence microscopy and anti-HSP60 antibody in SAT. Exercise had only marginal effects on HSP60 secretion and HSP60 autoantibody levels in plasma in both obese with and without diabetes. Physical exercise differentially alleviates cellular stress in obese adults with and without diabetes despite concomitant attenuation of the inflammatory response.

## Introduction

Obesity and type 2 diabetes (T2D) are global public health problems affecting both people’s quality of life and socioeconomics around the globe (1). The pathophysiology of these metabolic diseases is closely linked, with the resulting insulin resistance (IR) as the cause of several health comorbidities (2). IR has been demonstrated to be associated with various micro- and macrovascular complications (3). However, in obesity, the risk for these complications differs among individuals, as a significant proportion of obese people are metabolically healthy (4). Thus, the degree of metabolic dysregulation is a determinant of future complications in obese people.

The heat shock response (HSR) is a major stress adaptation mechanism that prevents insults to tissues, through a set of highly conserved proteins called heat shock proteins (HSPs) (5, 6). Some members of HSP are ubiquitously expressed, whereas others are expressed upon stress insults highlighting the critical role of HSP in maintaining cellular homeostasis. HSP can also be released into the circulation and exert an immune-stimulatory effect by interacting with pattern recognition receptors, such as toll-like receptors, and consequently activate the host inflammatory response (7, 8). Previous research demonstrated that HSR is attenuated in patients with T2D; in particular, heat shock protein 72 (HSP72) expression was decreased in patients with diabetes (9). Moreover, HSP72 induction resulted in protective effects in humans with diabetes and diabetic animal models (10, 11). Specifically, HSP72 induction led to improved lipid accumulation in the liver and adipose tissue, reduced inflammatory signals, and improved insulin sensitivity. By contrast, we recently observed increased expression of major HSPs, including HSP72, in both adipose tissue and blood cells from obese people without diabetes (12). This finding suggests that in this population the HSR can resolve metabolic stresses attributable to obesity, thus highlighting differences in molecular pathophysiology between obese subjects with and without diabetes even though both conditions are associated with IR.

Another key member of the HSR, HSP60, is notable for its ability as mediator of immunity in several inflammatory diseases such as cancer, atherosclerosis, adjuvant arthritis, obesity, and diabetes (13, 14). HSP60 is mainly a mitochondrial chaperone, but its translocation to the cytosol and cell membrane and secretion into blood have been reported (15). Furthermore, the ability of HSPs to induce different stress-related responses according to their subcellular localization has been reported (16). It is reported that circulating HSPs can have immunostimulating or immunosuppressive effects, in an apparently contradictory effect, depending on the context and types of interacting partners (17–20). Accumulating evidence suggests that circulating HSP60 may contribute to cardiovascular disease associated with diabetes, supporting earlier observations regarding the association between HSP60 and atherosclerosis (13, 21). Recent findings demonstrated that autoimmunity to HSP60 contributes to metabolic dysregulation in a murine obesity model, which were partially reversed by HSP60 peptide treatment (22). In contrast, human HSP60 displayed protective effects against adjuvant arthritis and contributed to remission in juvenile idiopathic arthritis in humans (14). Likewise, another recent study revealed that HSP60 promotes tissue regeneration and wound healing by regulating inflammation in animal models such as db/db mice and zebrafish (23). Furthermore, HSPs, including HSP60 and its derived peptides, can protect allografts from Ischemia–reperfusion injury and improved graft survival through IL-10 induction (24). Finally, in a recent study morbid obese have displayed a sustainable decrease in circulating HSP60 levels after bariatric surgery intervention concomitantly with a decrease in CRP but not in IL-6 (25). However, the biological significance of extracellular HSP60 remains to be elucidated. Intracellular HSP60 has a complex function, given that it inhibits caspase-3 but facilitates the maturation of pro-caspase-3 to its active form (13). Furthermore, HSP60 is implicated in mitochondrial biogenesis, and this capacity to promote the folding of mitochondrial proteins appears crucial for its cytoprotective function (26).

Conversely, it was reported that HSP60 levels are decreased in the heart but increased in the kidneys and liver of diabetic rats, thus highlighting the tissue specificity of the alteration of HSP expression in diabetes (27). However, the effect of different degrees of adiposity and related IR on variations in intra- and extracellular HSP levels across individuals and its influence on metabolic diseases remain to be clarified. Therefore, this study was designed to investigate the status of HSP60 in obese subjects with and without diabetes and assess the effects of physical activity on its levels in these two groups.

## Materials and Methods

### Study Population

The study consisted of obese (30 kg/m^2^ ≤ BMI < 40 kg/m^2^) adult men (*n* = 120) and women (*n* = 110) (non-diabetes group, *n* = 138; diabetes group, *n* = 92). Informed written consent was obtained from all subjects before their participation in the study, which was approved by the Review Board of Dasman Diabetes Institute and conducted in line with principles of the Declaration of Helsinki. Participants who performed any physical exercise within the last 6 months prior to study entry and those with prior histories of major illness or the use of medications and/or supplements known to influence body composition or bone mass were excluded from the study. The physical, clinical, and biochemical characteristics of the participating subjects are shown in Table 1.

**Table 1:** Physical, clinical, and biochemical characteristics of the subjects at baseline.

	Obese non-diabetic(*n* = 138)	Obese diabetic(*n* = 92)	*p*
**Physical and clinical characteristics**			
Age (years)	45.76 ± 9.91	48.28 ± 8.17	0.16
Gender (M/F)	71/67	49/43	0.44
BMI (kg/m^2^)	33.82 ± 3.47	33.55 ± 2.94	0.59
PBF (%)	38.20 ± 5.35	37.02 ± 4.95	0.54
Waist (cm)	106.02 ± 12.00	108.88 ± 8.75	0.053
Hip (cm)	115.06 ± 12.64	113.96 ± 12.40	0.97
Resting HR (beats/min)	76.67 ± 11.01	86.01 ± 11.68	<0.0001
SBP (mmHg)	120.92 ± 13.04	121.88 ± 11.92	0.64
DBP (mmHg)	78.13 ± 8.58	78.70 ± 6.16	0.28
VO2 Max (mL/kg/min)	17.57 ± 4.70	15.89 ± 4.10	0.04
WBC10	6.65 ± 1.89	7.54 ± 1.91	0.0003
**Metabolic markers**			
Cholesterol (mmol/L)	5.21 ± 0.89	5.05 ± 1.16	0.22
HDL (mmol/L)	1.22 ± 0.36	1.11 ± 0.33	0.027
LDL (mmol/L)	3.35 ± 0.85	3.16 ± 1.24	0.09
TG (mmol/L)	1.45 ± 0.95	2.10 ± 1.74	0.0002
Glucose (mmol/L)	5.43 ± 0.47	9.82 ± 3.85	<0.0001
HbA1c (%)	5.76 ± 0.49	8.70 ± 1.93	<0.0001
Insulin (ng/mL)	3.82 ± 1.94	4.22 ± 2.04	0.26
HOMA-IR	0.96 ± 0.55	1.60 ± 0.88	<0.0001
C-peptide (g/mL)	1.88 ± 1.13	1.87 ± 1.25	0.91
Glucagon (ng/mL)	0.16 ± 0.04	0.18 ± 0.05	0.11
GIP (ng/mL)	0.76 ± 0.55	0.92 ± 0.61	0. 13
GLP-1 (ng/mL)	0.27 ± 0.05	0.29 ± 0.04	0.002
Leptin (ng/mL)	9.10 ± 6.25	8.66 ± 3.99	0.74
**Inflammatory markers**			
IL-1b (pg/L)	8.29 ± 3.28	7.82 ± 1.94	0.96
IL-6 (pg/mL)	17.30 ± 6.24	17.16 ± 4.87	0.71
IL-10 (pg/mL)	28.47 ± 20.05	33.02 ± 27.87	0.50
IP-10 (pg/mL)	571 ± 418	689 ± 617	0.54
TNF-α (pg/mL)	127 ± 40	123 ± 31	0.65
hsCRP (μg/mL)	5.38 ± 3.91	6.36 ± 4.52	0.24

*Data are presented as the mean ± SD. HR, heart rate; SBP, systolic blood pressure; DBP, diastolic blood pressure; VO_2_ Max, maximum oxygen consumption; HDL, high-density lipoprotein; LDL, low-density lipoprotein; TG, triglyceride; HbA1c, hemoglobin A1c; hsCRP, high-sensitivity CRP*.

### Exercise Protocol and Anthropometric Measurements

All eligible subjects were enrolled in a supervised exercise program at the Fitness and Rehabilitation Center (FRC) of the Dasman Diabetes Institute as previously reported (28). Briefly, prior to exercise, each subject underwent an initial physical assessment to determine his or her maximum heart rate (max HR) as well as his or her response to aerobic exercise as measured by the maximum oxygen consumption (VO_2_ Max). The exercise regimen involved a combination of moderate-intensity aerobic exercise and resistance training using either a treadmill or stationary bicycle. Each exercise session included 10-min warm-up and cooldown steps at 50–60% max HR and 40 min of the prescribed exercise program at 65–80% max HR. For the duration of the 3-month period, participants exercised three times per week. All sessions were supervised by qualified fitness professionals at FRC to ensure that participants reached and maintained the recommended HR range. Anthropometric measurements were taken at baseline and after 3 months of exercise, and the intensity and duration of exercise as well as blood pressure were recorded for each session. Whole-body composition was determined using an IOI 353 Body Composition Analyzer (Jawon Medical, Seoul, Korea).

### Blood and Tissue Sampling

Venous peripheral blood and subcutaneous adipose tissue (SAT) biopsies were obtained at baseline and after 3 months of exercise. Plasma samples were prepared using EDTA Vacutainer tubes, aliquoted, and stored at −80°C. Subcutaneous superficial adipose tissue biopsies (approximately 0.5 g) were obtained from the periumbilical area *via* surgical biopsy after local anesthesia. Once removed, each biopsied tissue was rinsed in cold PBS, divided into four pieces, and stored appropriately until assayed.

### Blood Inflammatory and Metabolic Markers

Glucose and lipid profiles were measured using a Siemens Dimension RXL chemistry analyzer (Diamond Diagnostics, Holliston, MA, USA). Hemoglobin A1c (HbA1c) levels were determined using the Variant™ device (BioRad, Hercules, CA, USA). Insulin and high-sensitivity CRP (hsCRP) levels were determined using a Mercodia Insulin ELISA Kit (Mercodia AB, Uppsala, Sweden) and an hsCRP ELISA kit (Biovendor, Asheville, NC, USA), respectively. Plasma levels of inflammatory and metabolic markers were measured using bead-based multiplexing technology on a Bioplex-200 system (BioRad). All of the aforementioned assays were performed according to the manufacturers’ instructions. The Homeostatic Model Assessment of Insulin Resistance (HOMA-IR) index was calculated using the following formula: HOMA-IR = (glucose × insulin)/22.5.

### Immunohistochemistry (IHC) and Immunofluorescence (IF)

Formalin-fixed, paraffin-embedded SAT sections were used for IHC and IF investigations as described previously (12, 28). Anti-HSP60 (Enzo LifeSciences, Inc., Lausen, Switzerland), anti-TNF-α (Abcam, Inc., Cambridge, MA, USA), and anti-IL-6 antibodies (Novus Biologicals, LLC, Littleton, CO, USA) were used for IHC. Quantification of the IHC data was performed using ImageScope software version 11.1 (Aperio, Vista, CA, USA) as previously reported (12). For IF staining, tissue sections were incubated with an Alexa Fluor^®^ 488-conjugated anti-HSP60 antibody (Bioss Inc., Woburn, MA, USA). DAPI was used at 0.05% for nuclear staining. The sections were analyzed using a Zeiss LSM 710 confocal laser-scanning microscope, and fluorescent images of the representative areas of the adipose tissue were photographed using a × 40 objective.

### Quantitative Real time (qRT)-PCR

Total RNA was extracted from frozen adipose tissue using an RNeasy Lipid Tissue Mini Kit (Qiagen, Inc., Valencia, CA, USA). cDNA was synthesized from total RNA samples using High Capacity cDNA Reverse Transcription Kits (Applied Biosystems, Foster City, CA, USA). Conventional qRT-PCR was performed on a Rotor Gene Q-100 system using SYBR Green normalized to GAPDH (Qiagen). The relative gene expression between the groups was assessed using the ΔΔCT method (29), and GAPDH was used as internal control for normalization. Primers used for validation are displayed in Table S1 in Supplementary Material.

### Quantification of Circulating Proteins by ELISA

Plasma levels of HSP60 were measured by using a sandwich immunoassay EIA kit (ADI-EKS-600, Enzo, PA, USA). Plasma levels of anti-HSP60 IgG/A/M were measured using an ELISA kit (ADI-EKS-650, Enzo). Samples were diluted 1:2 before analysis for HSP60. After optimization, undiluted serum samples were used to measure anti-HSP60 IgG/IgA/IgM levels. All assays were performed according to the manufacturer’s instructions. Absorbance was measured at 450 nm on an H4 Synergy plate reader (Biotek, Winooski, VT, USA).

### Statistical Analysis

Statistical analyses were performed using SPSS software (v22.0; SPSS Inc., Chicago, IL, USA). Unless otherwise stated, all descriptive statistics for the variables in the study were reported as the mean ± SD. Normality tests were run to assess the data distribution. A parametric *t*-test was used for variables with normal distributions to assess the significance of differences in means between the groups before exercise, whereas the Mann–Whitney non-parametric *t*-test was used for the skewed variables. A paired *t*-test was used to determine the significance of differences in means inside non-diabetic and diabetic groups before and after exercise. To evaluate the effect of groups and exercise intervention as well as their combination, we conducted two-way repeated measures analysis of variance (ANOVA). Effect sizes and homogeneity for ANOVA outcomes were examined using partial eta-squared, Box’s *M* test and Levene’s test of equality. For all analysis, differences were considered statistically significant at *p* < 0.05.

## Results

### Baseline Characteristics of the Study Population and the Effects of Physical Exercise

The anthropometric, clinical, and metabolic characteristics of the subjects are summarized in Table 1. There were no significant differences between the two groups regarding gender, age, waist or hip circumference, BMI, percent body fat (PBF), and blood pressure. Subjects in the diabetes group had a significantly higher resting HR and a significantly lower VO_2__Max_ than those in the non-diabetes group. Concerning lipid profiles, the diabetes group had higher triglyceride (TG) levels but lower HDL levels, whereas total cholesterol and LDL levels were similar between the two groups. Although fasting blood glucose (FBG), HbA1c, and HOMA-IR values were significantly higher in the diabetes group, there was no difference between the two groups regarding serum insulin or C-peptide concentrations in the blood. Furthermore, GLP-1 levels were higher in the diabetes group (*p* = 0.02), whereas leptin, glucagon, and GIP levels were similar between the two groups. Finally, no significant difference was detected between the two groups concerning all inflammatory markers assayed (Table 1).

Physical exercise is considered the first-line non-pharmacologic treatment for preventing and managing lifestyle-related diseases. Our group and others have previously demonstrated the beneficial effects of physical exercise on the expression and secretion of stress proteins (12, 30). In this study, we performed a pairwise comparison of physical, clinical, and metabolic parameters in the diabetes and non-diabetes groups (*n* = 43, each) before and after physical exercise, the results of which are displayed in Tables 2 and 3, respectively. For the non-diabetes group, significant decreases were observed in adiposity markers (BMI, waist circumference, and PBF) after exercise (*p* ≤ 0.01). Likewise, we detected significant decreases in systolic and diastolic blood pressure (*p* < 0.01 and *p* < 0.05, respectively), along with an improvement of VO_2__Max_ (*p* < 0.001). Furthermore, physical exercise decreased glycemic index markers such as insulin, HOMA-IR, and C-peptide values (*p* < 0.01, *p* = 0.05, and *p* < 0.05, respectively) in addition to a significant decrease in GIP levels (*p* = 0.005). Finally, our results revealed a trend toward increase for some circulating inflammation markers after physical exercise (Table 2). In the diabetes group, physical exercise had limited effects on physical parameters, as only waist circumference and VO_2__Max_ were significantly improved (*p* < 0.05) (Table 3). However, superior improvements in metabolic markers were recorded in this group. Indeed, exercise significantly decreased metabolic markers such as cholesterol, HbA1c, C-peptide, glucagon, GIP, and GLP-1 levels (*p* < 0.05), whereas no effects were observed on inflammatory markers.

**Table 2:** Physical, clinical, and biochemical characteristics of obese subjects without diabetes before and after exercise.

	Before exercise(*n* = 43)	After exercise(*n* = 43)	*p*
**Physical and clinical characteristics**			
Age (years)	47.58 ± 9.44	–	
Gender (M/F)	23/20	–	
BMI (kg/m^2^)	33.08 ± 2.95	32.37 ± 3.63	0.0103
PBF (%)	36.72 ± 5.27	35.64 ± 5.56	0.0028
Waist (cm)	104.59 ± 10.28	101.21 ± 10.79	0.0040
Hip (cm)	112.97 ± 8.33	112.35 ± 8.95	0.51
Resting HR (beats/min)	73.65 ± 12.11	74.68 ± 11.41	0.97
SBP (mmHg)	121.74 ± 13.97	115.89 ± 8.50	0.0068
DBP (mmHg)	77.84 ± 8.81	75.18 ± 6.31	0.0229
VO_2_ Max (mL/kg/min)	18.61 ± 5.04	20.89 ± 5.84	0.0006
WBC10	6.52 ± 1.96	5.89 ± 1.74	0.42
**Metabolic markers**			
Cholesterol (mmol/L)	5.19 ± 0.78	5.39 ± 0.98	0.72
HDL (mmol/L)	1.23 ± 0.31	1.27 ± 0.41	0.507
LDL (mmol/L)	3.31 ± 0.72	3.56 ± 0.90	0.67
TG (mmol/L)	1.43 ± 0.63	1.37 ± 0.74	0.91
Glucose (mmol/L)	5.54 ± 0.37	5.66 ± 0.34	0.54
HbA1c (%)	5.78 ± 0.29	5.75 ± 0.31	0.208
Insulin (ng/mL)	4.07 ± 2.35	2.88 1.40	0.0048
HOMA-IR	1.03 ± 0.51	0.73 ± 0.20	0.051
C-peptide (μg/mL)	1.99 ± 1.35	1.40 ± 0.61	0.017
Glucagon (ng/mL)	0.15 ± 0.05	0.15 ± 0.05	0.52
GIP (ng/mL)	0.77 ± 0.51	0.41 ± 0.18	0.0051
GLP-1 (ng/mL)	0.26 ± 0.06	0.26 ± 0.08	0.81
Leptin (ng/mL)	9.95 ± 7.81	7.81 ± 5.98	0.24
**Inflammatory markers**			
IL-1b (pg/mL)	7.96 ± 3.30	9.10 ± 4.28	0.037
IL-6 (pg/mL)	17.84 ± 6.87	18.02 ± 10.07	0.106
IL-10 (pg/mL)	22.62 ± 12.04	32.91 ± 20.77	0.107
IP-10 (pg/mL)	578 ± 438	542 ± 264	0.72
TNF-α (pg/mL)	115 ± 34	131 ± 57	0.065
hsCRP (µg/mL)	4.35 ± 3.03	4.03 ± 3.59	0.43

*Data are presented as the mean ± SD. HR, heart rate; SBP, systolic blood pressure; DBP, diastolic blood pressure; VO_2_ Max, maximum oxygen consumption; HDL, high-density lipoprotein; LDL, low-density lipoprotein; TG, triglyceride; HbA1c, hemoglobin A1c; hsCRP, high-sensitivity CRP*.

**Table 3:** Physical, clinical, and biochemical characteristics of the obese subjects with diabetes before and after exercise.

	Before exercise(*n* = 43)	After exercise(*n* = 43)	*p*
**Physical and clinical characteristics**			
Age (years)	47.14 ± 7.96	–	
Gender (M/F)	24/19	–	
BMI (kg/m^2^)	33.24 ± 2.90	32.37 ± 2.88	0.074
PBF (%)	36.30 ± 5.06	35.04 ± 5.25	0. 171
Waist (cm)	108.91 ± 8.77	105.70 ± 7.43	0.013
Hip (cm)	111.34 ± 13.75	110.89 ± 6.73	0.41
Resting HR (beats/min)	87.15 ± 11.65	85.23 ± 10.40	0.49
SBP (mmHg)	122.00 ± 11.81	121.38 ± 12.78	0.63
DBP (mmHg)	78.75 ± 5.63	77.48 ± 5.91	0.35
VO_2_ Max (mL/kg/min)	16.88 ± 3.54	20.10 ± 4.53	0.042
WBC10	7.75 ± 2.00	7.44 ± 1.99	0.146
**Metabolic markers**			
Cholesterol (mmol/L)	5.06 ± 1.24	4.44 ± 0.88	0.014
HDL (mmol/L)	1.01 ± 0.26	1.02 ± 0.28	0.69
LDL (mmol/L)	3.30 ± 1.51	2.79 ± 0.86	0.086
TG (mmol/L)	1.86 ± 1.02	1.60 ± 0.69	0.96
Glucose (mmol/L)	10.08 ± 4.26	8.83 ± 3.21	0.35
HBA1C (%)	8.62 ± 1.96	7.54 ± 1.48	0.0012
Insulin (ng/mL)	4.09 ± 1.88	3.41 ± 2.18	0.158
HOMA-IR	1.56 ± 0.84	1.27 ± 0.65	0.105
C-peptide (μg/mL)	1.94 ± 1.05	1.44 ± 0.78	0.007
Glucagon (ng/mL)	0.18 ± 0.05	0.17 ± 0.04	0.0247
GIP (ng/mL)	0.93 ± 0.52	0.51 ± 0.24	0.0037
GLP-1 (ng/mL)	0.29 ± 0.04	0.28 ± 0.04	0.024
Leptin (ng/mL)	7.25 ± 3.39	6.92 ± 3.94	0.91
**Inflammatory markers**			
IL-1b (pg/mL)	7.63 ± 1.76	7.99 ± 1.81	0.35
IL-6 (pg/mL)	17.49 ± 4.93	17.49 ± 4.44	0.62
IL-10 (pg/mL)	33.18 ± 30.45	34.85 ± 24.78	0.56
IP-10 (pg/mL)	786 ± 670	735 ± 602	0.74
TNF-α (pg/mL)	118 ± 30	125 ± 24	0.68
hsCRP (μg/mL)	6.35 ± 4.09	5.19 ± 3.47	0.25

*Data are presented as mean ± SD. HR, heart rate; SBP, systolic blood pressure; DBP, diastolic blood pressure; VO_2_ Max, maximum oxygen consumption; HDL, high-density lipoprotein; LDL, low-density lipoprotein; TG, triglyceride; HbA1c, hemoglobin A1c; hsCRP, high-sensitivity CRP*.

To further assess the effect of diabetes and exercise intervention as well as their combined effect, we used two-way ANOVA with repeated measures analysis. As displayed in Table 4, the separate effects of exercise and diabetes were in agreement with the results obtained using paired *t*-test in particular for adiposity and glycemic index markers. Interestingly, with ANOVA analysis, the exercise significantly increased circulating inflammatory markers (IL-1β, IL-6, TNF-α, and IL-10) and decreased WBC, while diabetes displayed significant effect on HR and TNF-α. The combined effect of both disease and intervention, however, did not show any significance for all analyzed markers except a borderline significance for VO_2__Max_.

**Table 4:** Effect of diabetes and exercise on physical, clinical, and biochemical characteristics of the obese subjects using two-way ANOVA analysis.

		Non-diabetic (*n* = 43)	Diabetic (*n* = 43)	Exercise effect	Diabetes effect	Exercise x diabetes effect
Age (years)		47.58 ± 9.44	47.14 ± 7.96	Sig.	Sig.	Sig.

Gender (M/F)		23/20	24/19			

BMI(kg/m^2^)	Before	33.08 ± 2.95	33.24 ± 2.90	0.002	0.748	0.405
	After	32.37 ± 3.63	32.37 ± 2.88			

PBF (%)	Before	36.72 ± 5.27	36.30 ± 5.06	0.010	0.332	0.853
	After	35.64 ± 5.56	35.04 ± 5.25			

Waist (cm)	Before	104.59 ± 10.28	108.91 ± 8.77	0.005	0.249	0.403
	After	101.21 ± 10.79	105.70 ± 7.43			

Hip (cm)	Before	112.97 ± 8.33	111.34 ± 13.75	0.628	0.270	0.258
	After	112.35 ± 8.95	110.89 ± 6.73			

Resting HR (beats/min)	Before	73.65 ± 12.11	87.15 ± 11.65	0.186	0.011	0.649
	After	74.68 ± 11.41	85.23 ± 10.40			

SBP (mmHg)	Before	121.74 ± 13.97	122.00 ± 11.81	0.331	0.389	0.198
	After	115.89 ± 8.50	121.38 ± 12.78			

DBP (mmHg)	Before	77.84 ± 8.81	78.75 ± 5.63	0.221	0.907	0.431
	After	75.18 ± 6.31	77.48 ± 5.91			

VO2 Max (mL/kg/min)	Before	18.61 ± 5.04	16.88 ± 3.54	0.000	0.480	0.056
	After	20.89 ± 5.84	20.10 ± 4.53			

WBC10	Before	6.52 ± 1.96	7.75 ± 2.00	0.003	0.195	0.973
	After	5.89 ± 1.74	7.44 ± 1.99			

Cholesterol (mmol/L)	Before	5.19 ± 0.78	5.06 ± 1.24	0.240	0.099	0.297
	After	5.39 ± 0.98	4.44 ± 0.88			

HDL (mmol/L)	Before	1.23 ± 0.31	1.01 ± 0.26	0.870	0.134	0.643
	After	1.27 ± 0.41	1.02 ± 0.28			

LDL (mmol/L)	Before	3.31 ± 0.72	3.30 ± 1.51	0.336	0.121	0.398
	After	3.56 ± 0.90	2.79 ± 0.86			

TG (mmol/L)	Before	1.43 ± 0.63	1.86 ± 1.02	0.573	0.327	0.836
	After	1.37 ± 0.74	1.60 ± 0.69			

Glucose (mmol/L)	Before	5.54 ± 0.37	10.08 ± 4.26	0.827	0.013	0.692
	After	5.66 ± 0.34	8.83 ± 3.21			

HbA1c (%)	Before	5.78 ± 0.29	8.62 ± 1.96	0.017	0.012	0.630
	After	5.75 ± 0.31	7.54 ± 1.48			

Insulin (ng/mL)	Before	4.07 ± 2.35	4.09 ± 1.88	0.027	0.811	0.381
	After	2.88 1.40	3.41 ± 2.18			

HOMA-IR	Before	1.03 ± 0.51	1.56 ± 0.84	0.030	0.047	0.933
	After	0.73 ± 0.20	1.27 ± 0.65			

C-peptide (μg/mL)	Before	1.99 ± 1.35	1.94 ± 1.05	0.310	0.144	0.540
	After	1.40 ± 0.61	1.44 ± 0.78			

Glucagon (ng/mL)	Before	0.15 ± 0.05	0.18 ± 0.05	0.459	0.228	0.292
	After	0.15 ± 0.05	0.17 ± 0.04			

GIP (ng/mL)	Before	0.77 ± 0.51	0.93 ± 0.52	0.005	0.238	0.937
	After	0.41 ± 0.18	0.51 ± 0.24			

GLP-1 (ng/mL)	Before	0.26 ± 0.06	0.29 ± 0.04	0.482	0.963	0.377
	After	0.26 ± 0.08	0.28 ± 0.04			

Leptin (ng/mL)	Before	9.95 ± 7.81	7.25 ± 3.39	0.301	0.571	0.215
	After	7.81 ± 5.98	6.92 ± 3.94			

IL-1b (pg/mL)	Before	7.96 ± 3.30	7.63 ± 1.76	0.010	0.156	0.478
	After	9.10 ± 4.28	7.99 ± 1.81			

IL-6 (pg/mL)	Before	17.84 ± 6.87	17.49 ± 4.93	0.012	0.171	0.263
	After	18.02 ± 10.07	17.49 ± 4.44			

IL-10 (pg/mL)	Before	22.62 ± 12.04	33.18 ± 30.45	0.005	0.572	0.480
	After	32.91 ± 20.77	34.85 ± 24.78			

IP-10 (pg/mL)	Before	578 ± 438	786 ± 670	0.567	0.376	0.674
	After	542 ± 264	735 ± 602			

TNF-α (pg/mL)	Before	115 ± 34	118 ± 30	0.019	0.034	0.215
	After	131 ± 57	125 ± 24			

hsCRP (μg/mL)	Before	4.35 ± 3.03	6.35 ± 4.09	0.681	0.402	0.968
	After	4.03 ± 3.59	5.19 ± 3.47			

*Data are presented as the mean ± SD. HR, heart rate; SBP, systolic blood pressure; DBP, diastolic blood pressure; VO_2_ Max, maximum oxygen consumption; HDL, high-density lipoprotein; LDL, low-density lipoprotein; TG, triglyceride; HbA1c, hemoglobin A1c; hsCRP, high-sensitivity CRP. Sig (significant) of two-way ANOVA*.

### HSP60 Differentially Expressed and Modulated by Physical Exercise in Obese Subjects with and without Diabetes

**Figure 1:**
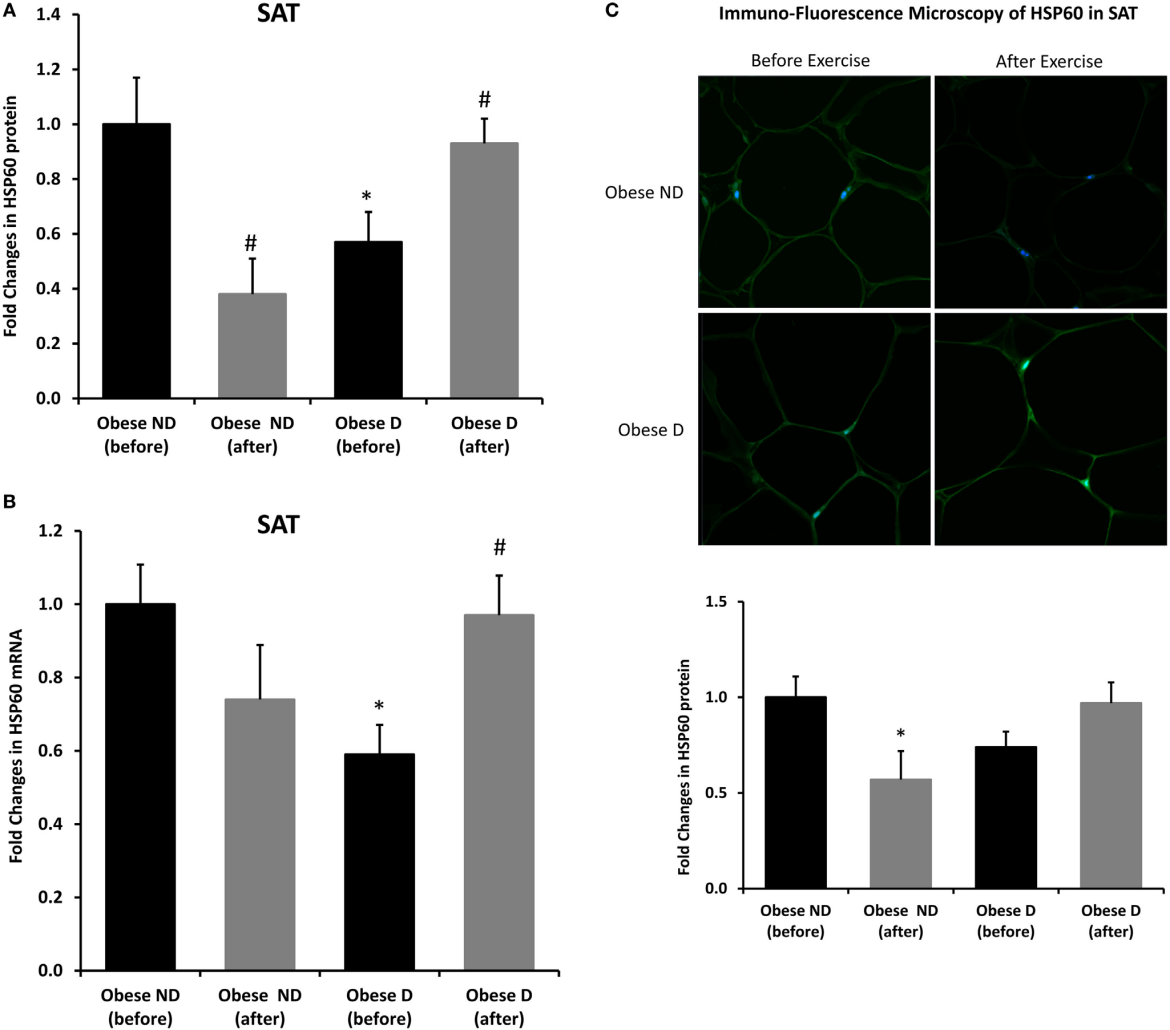
Decreased expression of HSP60 and modulation of its expression by exercise in the subcutaneous adipose tissue (SAT) of obese subjects with diabetes. **(A)** Immunohistochemical analysis of HSP60 expression in SAT sections from obese people without (ND) and with diabetes (D) before and after a 3-month physical exercise intervention (*n* = 10 for each group). **(B)** mRNA levels were measured by quantitative real-time PCR using SAT from obese subjects without (ND) and with diabetes (D) (*n* = 10 for each group) and normalized using GAPDH. Data are presented as fold changes in obese people with diabetes compared with the findings in the counterparts without diabetes. **(C)** Representative confocal immunofluorescence images illustrating HSP60 expression and localization in SAT from obese people without and with diabetes (*n* = 3 for each group). Densitometry quantification of the staining in SAT slides was performed as mentioned in Section “Materials and Methods.” The *p*-value was determined using the Mann–Whitney test for comparisons between the groups and using a paired *t*-test for intragroup comparisons before and after exercise. * denotes *p* < 0.05 between the diabetes and non-diabetes groups, and ^#^ denotes *p* < 0.05 between before and after exercise.

**Figure 2:**
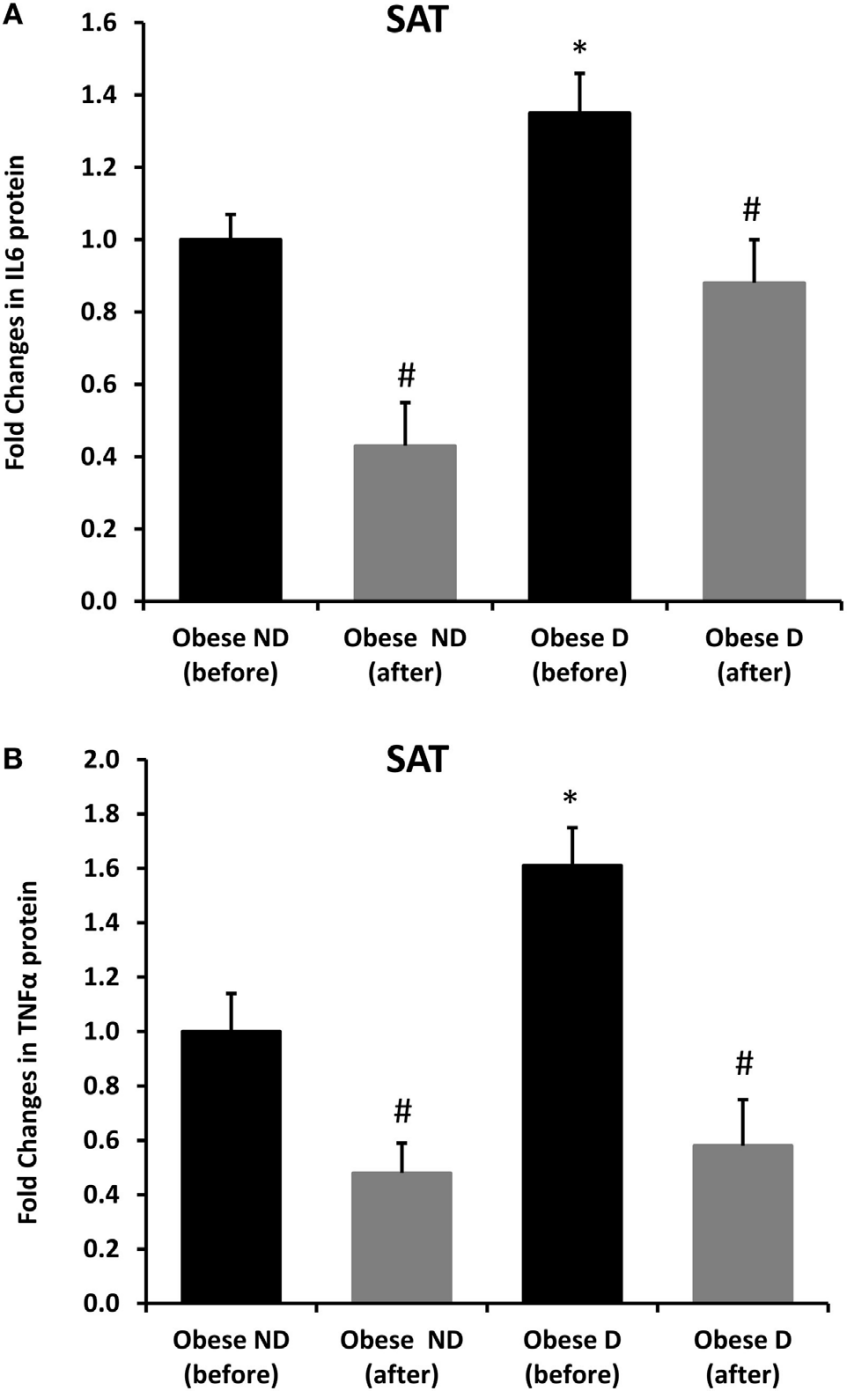
Increased inflammation and its modulation by exercise in the subcutaneous adipose tissue (SAT) of obese subjects with diabetes. Immunohistochemical analysis of **(A)** IL-6 and **(B)** TNF-α expression in SAT sections from obese people without (ND) and with diabetes (D) before and after 3 months of physical exercise (*n* = 10 for each group). Data are presented as fold changes in the diabetes group compared with the findings in the non-diabetes group. The *p*-value was determined using the Mann–Whitney test for comparisons between the diabetes and non-diabetes groups and using a paired *t*-test for intragroup comparisons before and after exercise. * denotes *p* < 0.05 between the diabetes and non-diabetes groups, and ^#^ denotes *p* < 0.05 between before and after exercise.

Decreased expression of HSPs, especially HSP72, has been widely reported in both human and animal models of IR and diabetes. By contrast, in previous work using SAT biopsies and PBMCs from obese patients without diabetes and their lean controls, we unexpectedly observed significant increases in HSP expression in obese subjects (12). As HSP60 is also involved in inflammation, a hallmark of diabetes, we assessed HSP60 expression levels in obese adults with and without diabetes. Our results revealed decreased expression of HSP60 at the protein (Figure 1A) and mRNA levels (Figure 1B) in SAT along with decreased HSP72 expression (Figure S1 in Supplementary Material) in the diabetes group. Using SAT and confocal IF microscopy, differential HSP60 patterns were confirmed between the groups (Figure 1C). Interestingly, the downregulation of HSP60 in adults with diabetes was concomitant with the increased expression of the tissue inflammatory cytokines produced by macrophage upon TLR or Th1 activation, IL-6 and TNF-α, as shown in Figure 2. HSP60 levels in blood serum were lower in the diabetes groups, whereas HSP60 autoantibody levels did not significantly differ between the two groups.

**Figure 3:**
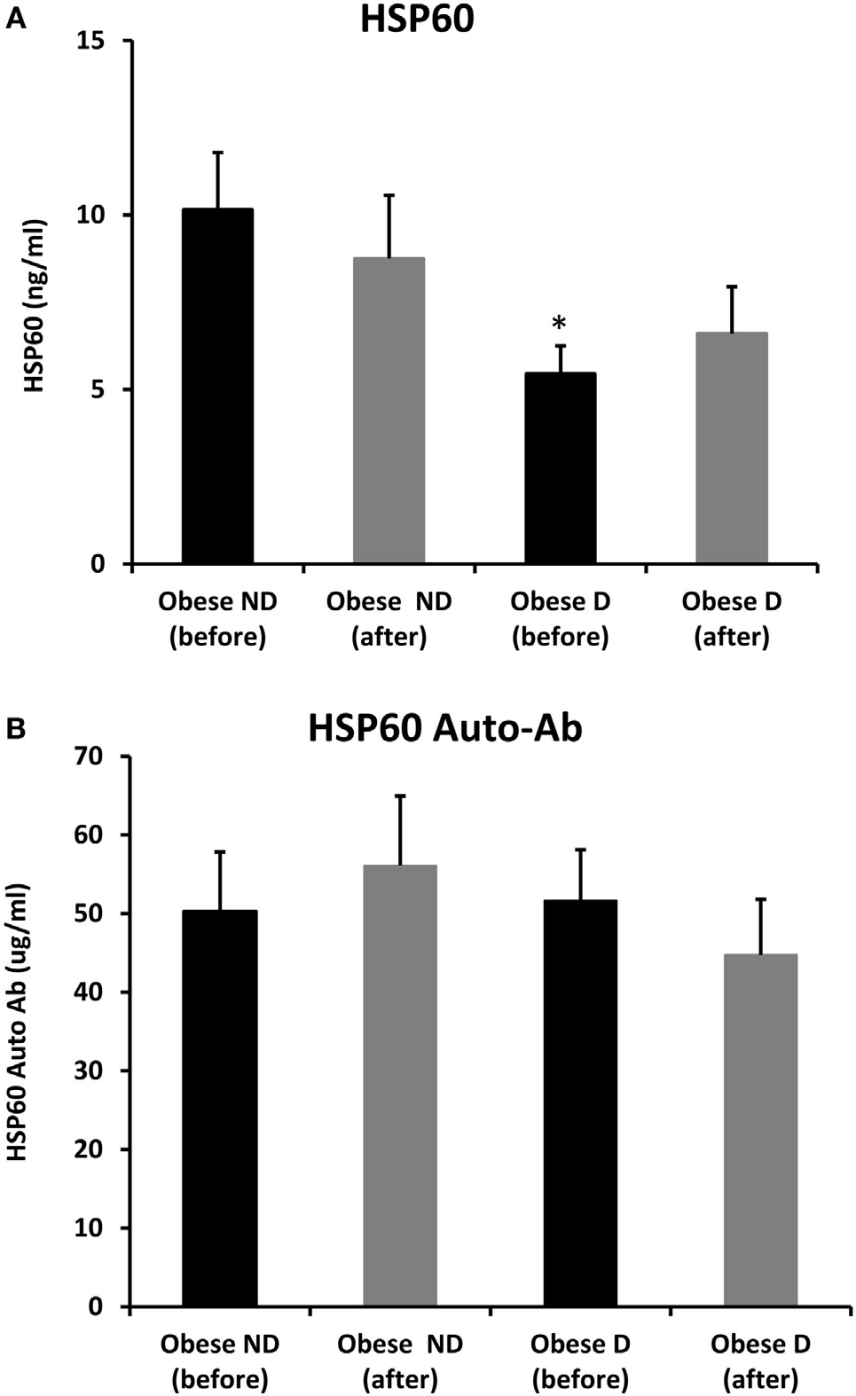
Secretion of HSP60 and HSP60 autoantibodies into blood. Circulating levels of **(A)** HSP60 protein and **(B)** HSP60 auto-Abs were measured by ELISA using plasma samples from obese people without (ND) and with diabetes (D) before and after a 3-month physical exercise intervention (*n* = 43 for each group). The *p*-value was determined using the Mann–Whitney test for comparisons between the diabetes and non-diabetes groups and using a paired *t*-test for intragroup comparisons before and after exercise. * denotes *p* < 0.05 between the diabetes and non-diabetes groups.

We further examined the effects of physical exercise on the expression and secretion of HSP60, and our results illustrated that exercise differentially affected HSP60 expression depending on the presence of diabetes. Indeed, HSP60 levels were increased in the diabetes group together with an increase in HSP72 levels, whereas clear decreases in IL-6 and TNF-α levels were noted in this group. However, an opposite pattern was observed in the non-diabetes group for HSP60 and HSP72, in addition to a decrease in inflammatory marker levels (Figures 1 and 2). Similarly, confocal IF microscopy confirmed the differential effect of physical exercise on HSP60 expression between the two groups. Finally, our physical exercise protocol did not significantly change the levels of circulating HSP60 and its autoantibodies in either study group, as shown in Figure 3. It is worth noting that the expression pattern of HSP60 was not related to gender as both males and females have shown similar trends for HSP60 levels in the SAT as well as in the blood before and after exercise intervention (data not shown).

## Discussion

Obese patients with diabetes have increased risks of morbidity and mortality compared with their non-diabetic counterparts, some of whom are metabolically healthy (31). HSP60 is a key protein involved in the crosstalk between metabolic stress and inflammation, as it participates in both the HSR and pro-inflammatory/anti-inflammatory processes. The aim of the present study was to assess the differential expression of HSP60 in the adipose tissue of obese adults with and without diabetes and its changes in response to physical exercise. Our main findings were as follows: (i) HSP60 levels were decreased in the diabetes group together with increased inflammatory and glycemic marker levels and lower fitness compared with the findings in the non-diabetes group; and (ii) moderate physical exercise differentially modulated HSP60 and HSR but attenuated inflammation in both groups, suggesting different beneficial effects between obese patients with and without diabetes.

The status of the HSR and differential expression of its major components between obese people with and without diabetes remain to be investigated, especially in adipose tissue. We previously reported that obesity increased the expression of HSR components in obese people without diabetes compared with their levels in normal-weight controls (12). Recently, we demonstrated that GRP78, another heat shock-induced chaperone participating in the unfolded protein response (UPR), was upregulated in obese people without diabetes, but its upregulation was more pronounced in obese people with diabetes (30). By contrast, other groups previously observed decreasing levels of HSPs in obese people (9, 11, 32, 33). However, these studies mainly used muscle tissue from obese people with diabetes and animal models, thus highlighting the possibility of tissue-specific expression patterns. In this study, in agreement with other findings, we found that HSP72 levels in SAT are clearly attenuated with a concomitant decrease in HSP60 expression in obese people with diabetes compared with the findings in obese people without diabetes. Another study illustrated that the ratio of HSP60 levels between visceral adipose tissue and SAT was higher in obese people with diabetes than in obese people without diabetes (34). This finding reflected either a decrease in HSP60 levels in SAT or an increase in its levels in visceral adipose tissue. Interestingly, *in vivo* and *in vitro* heat treatment differentially affected HSP expression patterns across adipose tissue depots, underscoring the fact that the HSR is also depot-specific (35). Moreover, Marker and colleagues (34) used primary adipocytes, and thus, differences in experimental procedures and types of samples, in this case biopsies versus primary cell culture, must be considered when attempting to reach a consensus concerning HSR response signaling. Moreover, our results indicated that blood HSP60 levels were lower in obese people with diabetes than in their counterparts without diabetes (Figure 3), in line with a previous report suggesting that lower blood HSP60 levels were associated with an increased diabetes risk in male patients (36). In our current study, we included both sexes, and our controls were obese people without diabetes. This attenuation in HSP60 expression and secretion into blood in obese people with diabetes might have resulted from chronic glucolipotoxicity rather than changes in insulin secretion. Indeed, our two study groups exhibited similar levels of insulin secretion markers (insulin and C-peptide), whereas people with diabetes exhibited higher glucose and TG levels despite receiving treatment for the disease. In support of this finding, we further compared HSP60 expression levels in SAT from lean people with and without diabetes using samples available from our previous study (12). Our results revealed that HSP60 levels were attenuated in lean subjects with diabetes along with increased expression of the inflammatory markers IL-6 and TNF-α, as observed in our obese subjects (Figure S2 in Supplementary Material). The fact that despite the clear increase in the levels of inflammatory markers in the adipose tissue of diabetic subjects, we have not observed significant difference in soluble inflammatory markers in the blood might be related to the treatment taken by the diabetic, known to impact cellular stress and inflammation (37). The WBC levels, however, were significantly higher in diabetic obese (Table 1) which supports further the hypothesis that inflammation is primarily cellular.

In obesity, an increased HSR is an adaptive response to chronic stress concomitant with increased local inflammation in SAT but not in the circulation (12). Similarly, obesity-induced inflammation could be an initial protective and adaptive mechanism in response to fat storage. Accordingly, inflammation is considered a catabolic process facilitating energy expenditure (38). In obese people with diabetes, however, the evidence illustrates that inflammation is worsened (39). This was explained by persistent oxidative stress due to glucolipotoxicity, hormone dysregulation, and inflammation, leading to downregulation of the HSR and the transcription factor HSF1 through a diverted UPR (40). In line with this finding, we previously reported an increased UPR in obese people with diabetes compared with the findings in their counterparts without diabetes (31). In this context, the association between HSP60 and diabetes is complex. Indeed, several lines of evidence suggest that HSP60 induces both pro-inflammatory and anti-inflammatory cytokines (41). It was reported that when HSP60 acts as a pro-inflammatory mediator, it plays a role in unresolved vascular inflammation, which is strongly associated with diabetes, thus highlighting the regulatory role of HSP60 in modulating the inflammatory processes in diabetes and linking mitochondrial stress to inflammation. Furthermore, reduced levels of HSP60 in diabetic patients might be reflective of lower mitochondrial content in adipose tissue and thus less mitochondrial biogenesis, required for adipogenesis and lipid metabolism. In support of this hypothesis, diabetic (db/db) mice displayed lower HSP60 and mitochondrial capacity than obese (ob/ob) mice and which were corrected by rosiglitazone, PPARγ agonist that alleviates IR and lowers glucose levels in type 2 diabetic rodents (42, 43) as well as in human patients (44). Finally, and due to its broad function, HSP60 may have a direct role in the development of IR as reported in mice with heterozygous deletion of HSP60, which displayed IR and reduced mitochondrial capacity, aside with increased inflammation (45).

Several observational studies reported a marked reduction in the incidence of diabetes among physically active individuals, suggesting that a healthy lifestyle remains an important non-pharmacologic intervention for preventing diabetes (46). One of the beneficial effects of exercise is the modulation of inflammation and metabolic stress (47). Thus, understanding the effect of exercise on the crosstalk between the HSR and inflammation in obesity and diabetes would clarify its molecular rationale. From this perspective, we investigated the effect of 3 months of exercise on HSP60 expression in our study population, and our results interestingly demonstrated that HSP60 expression was differentially modulated in SAT depending on the presence of diabetes. Our previous study revealed that in obese people, HSP expression was decreased relative to that in normal-weight controls (12). In the current study, we confirmed our previously published results, specifically for HSP60 and HSP72, using obese people without diabetes as a control group. However, in obese subjects with diabetes, exercise increased HSP60 and HSP72 levels. This upregulation was concomitant with decreased inflammation in the SAT of both groups due to the exercise intervention. The hypothesis regarding whether this differential response was due to greater compliance with the physical exercise protocol in one group or the inability of the other group to appropriately respond to the exercise training program was eliminated, as obviously, the effect was opposite and our exercise protocol was similarly prescribed to both groups under the supervision of experts at our FRC. Thus, the differential effects of physical exercise between the two groups might be explained by differences in metabolic flexibility and adaptation between the groups. Indeed, it was previously reported that the metabolism of free fatty acids (FFAs) during physical exercise was different between obese people with and without diabetes, as the utilization of plasma FFAs was reduced in the latter group (48). Moreover, people with diabetes exhibit increased flux of FFAs and glucose, which is associated with the excessive production of reactive oxygen species in adipocytes (49). These effects might lead to a decrease in the differentiation capacity of preadipocytes in subjects with diabetes, as previously reported (50), and thus a reduced response to physical exercise. Furthermore, we observed that exercise more effectively improved the expression of molecular markers of inflammation and metabolism in obese people without diabetes even though no major change in body weight was observed in either group. The fact that ANOVA analysis displayed significant increase in circulating inflammatory markers does not contradict the decreased levels of IL-6 and TNF-α in SAT. An initial increase of those circulating cytokines due to exercise has been suggested to be an adaptive process to exercise stress and highlighting the good side of a subclinical inflammation (38, 51). The beneficial effects of exercise are further supported by the decreased levels of WBC, known to be increased in diabetic and CVD subjects as previously reported (52).

**Figure 4:**
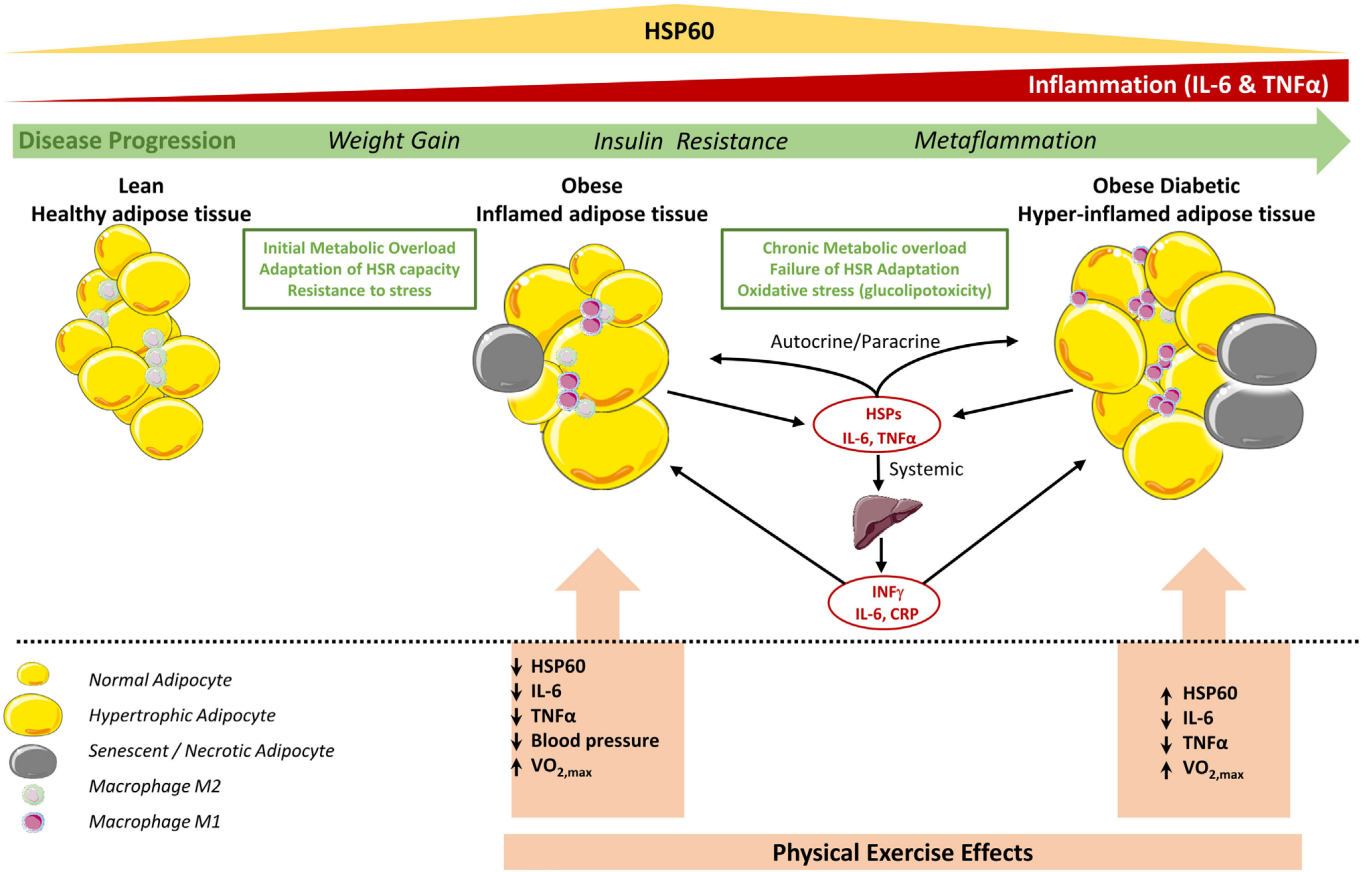
Status of HSP60 in subcutaneous adipose tissue (SAT) of obese subjects with and without diabetes and its modulation by physical exercise. In adipose tissue of lean subjects, most resident macrophages are M2 phenotype that contribute to insulin sensitivity. Metabolic overload and lack of physical activity increase body weight, hypertrophy of adipocytes, and number of M1 macrophages, which increase the secretion of pro-inflammatory cytokines such as TNF-α, IL-6 leading to obese inflamed adipose tissue. This contribute to the chronic subclinical metaflammation causing insulin resistance locally and probably in liver that amplifies the inflammation by secreting other pro-inflammatory mediators including IFNγ (53). At this stage, the HSR, in particular HSP60, levels are increased to cope with this cellular stress. However, in diabetic obese this metaflammation process is amplified due to high oxidative stress, which decreases mitochondrial function and HSP60 levels and finally a failure to control such hyper-inflamed adipose tissue. Regular physical exercise intervention decreases stress levels and inflammation in the adipose tissue for both diabetic and non-diabetic obese. While HSR is consequently decreased in non-diabetic obese subjects, in diabetic subjects, HSR and thus HSP60 are increased which might reflect an increase in mitochondrial capacity to reduce excessive metabolic stress. Cell pictures were adapted from Servier Medical Art.

As summarized in Figure 4, progression from a normal healthy status toward obesity and subsequently diabetes, increased fat accumulation, and metabolic dysregulation appears to be associated with the coordinated upregulation of the HSR and immune response in non-diabetic obese toward the development of an adaptive mechanism to cope with increased cellular stress. This HSR pattern is however reversed in diabetes, leading to an impaired response to exercise. A potential explanation of this differential effect is as follows: (i) in the case of obese without diabetes, exercise intervention has decreased the stress load on the SAT and the overall body and thus the HSR levels are attenuated, whereas (ii) in the case of obese with diabetes, the HSR as reflected by HSP60 is enhanced to cope with the persistent cellular dysregulated status despite the apparent decreased inflammation.

Another potential explanation of the observed HSR in adipose tissue of obese subjects with diabetes would be linked to cell senescence and necrosis (Figure 4). For instance, those processes are known to amplify inflammation through attracting more monocytes and pro-inflammatory mediators into the SAT. It was also reported that adipose tissue of obese and diabetic patients display both compromised HSR in adipocytes as well as in hepatocytes where adipose tissue displayed cellular senescence that spreads to all the metabolic tissues thereby determining a failure to resolve inflammation (40, 54). Furthermore, our previous observation that HSPs expression in obese subjects was "unexpectedly" increased in relation to lean volunteers (12) might be just a question of timing context as the HSR is enhanced when the tissues are under homeostatic-threatening situations (early stages of T2DM in obese people) but this is progressively reversed with time or lifestyle intervention.

On the other hand and as expected, HbA1c levels were higher in obese subjects with diabetes (Tables 1–3) but the moderate exercise program was not able to reverse these levels. Indeed, HOMA-IR values, despite being higher in those subjects, indicated just a moderate level of IR (ranges: 0.84–3.27 and 0.81–1.88, in subjects with diabetes and without diabetes, respectively). In this regard, exercise reduced HOMA-IR levels in subjects without diabetes but not in those with diabetes (ranges after exercise: 0.38–1.08 and 0.81–2.66, respectively). These observations can be explained by the fact that there is great variability between different geographic areas in the threshold of HOMA-IR levels to define IR and that HOMA-IR does not adequately predict IR in all individuals, in particular, with confirmed diabetes. Furthermore, it is reported that HOMA-IR and insulin action do not clearly correlate, particularly in individuals with impaired glucose tolerance (55–57). Furthermore, the hypothesis that our subjects with diabetes were not so metabolically jeopardized could be ruled out as all our diabetic subjects were clinically confirmed with diabetes and most of them already for more than 5 years.

Finally, despite the clear differential HSR patterns between obese people with and without diabetes, our study had some limitations including the lack of access to visceral adipose tissue or hepatic function markers. Indeed, these tissues are more reflective of metabolic events, and people with diabetes are known to have more visceral and intramuscular fat than those without diabetes (58). Another limitation of our study was the absence of any diet intervention, which might have increased the efficacy of physical exercise. However, our subjects were instructed to maintain a stable diet during the 3-month exercise program, but we did not monitor their compliance. Moreover, we chose to study the direct effects of exercise alone because we believe that moderate exercise is an attractive behavioral approach to improve global health without drastic diet restriction. Further cellular work is also warranted to elucidate in details the source and the function of HSP60 in the SAT.

In summary, our data illustrated that obese subjects with diabetes had decreased expression and secretion of HSP60. This decrease in expression was reverted by physical exercise in parallel with decreased expression of inflammatory markers in SAT despite marginal changes in BMI. Our results provide further molecular evidence of the beneficial effects of physical exercise for restoring cellular stress defenses through improving HSR in diabetes.

## Ethics Statement

Informed written consent was obtained from all subjects before their participation in the study, which was approved by the Review Board of Dasman Diabetes Institute and conducted in line with principles of the Declaration of Helsinki.

## Author Contributions

AK, MD, and AT designed the study. AK and AT wrote the manuscript. AK, JA, MD, and AT supervised data collection and analysis. AK, MD, and AT revised the manuscript. SK, PC, and SW participated in data collection and analysis.

## Conflict of Interest Statement

The authors declare that the research was conducted in the absence of any commercial or financial relationships that could be construed as a potential conflict of interest.

## Supplementary Material

Figure S1 Decreased expression of HSP72 and its modulation by exercise in the subcutaneous adipose tissue (SAT) of obese subjects with diabetes. Immunohistochemical analysis of HSP72 expression in SAT sections from obese people without (ND) and with diabetes (D) before and after a 3-month physical exercise intervention (*n* = 10 for each group). Data are presented as fold changes in the diabetes group compared with that in the non-diabetes group. The *p*-value was determined using the Mann–Whitney test for comparisons between the diabetes and non-diabetes groups and using a paired *t*-test for intragroup comparisons before and after exercise. * denotes *p* < 0.05 between the diabetes and non-diabetes groups, and ^#^ denotes *p* < 0.05 between before and after exercise.

Figure S2 Expression of HSP60, IL-6, and TNF-α in the subcutaneous adipose tissue (SAT) of lean subjects with diabetes. Representative confocal immunofluorescence images illustrating HSP60 **(A)**, IL-6 **(B)**, and TNF-α **(C)** expression and localization in SAT from lean people with and without diabetes (*n* = 3 for each group). Quantification of the staining in SAT slides was performed as mentioned in Section “Materials and Methods.”
