# Supplementary figures and images for: Physical Exercise Enhanced Heat Shock Protein 60 Expression and Attenuated Inflammation in the Adipose Tissue of Human Diabetic Obese
Source: Front Endocrinol (Lausanne). 2018 Feb 6;9:16. doi: 10.3389/fendo.2018.00016 (PMC5808138; doi:10.3389/fendo.2018.00016)

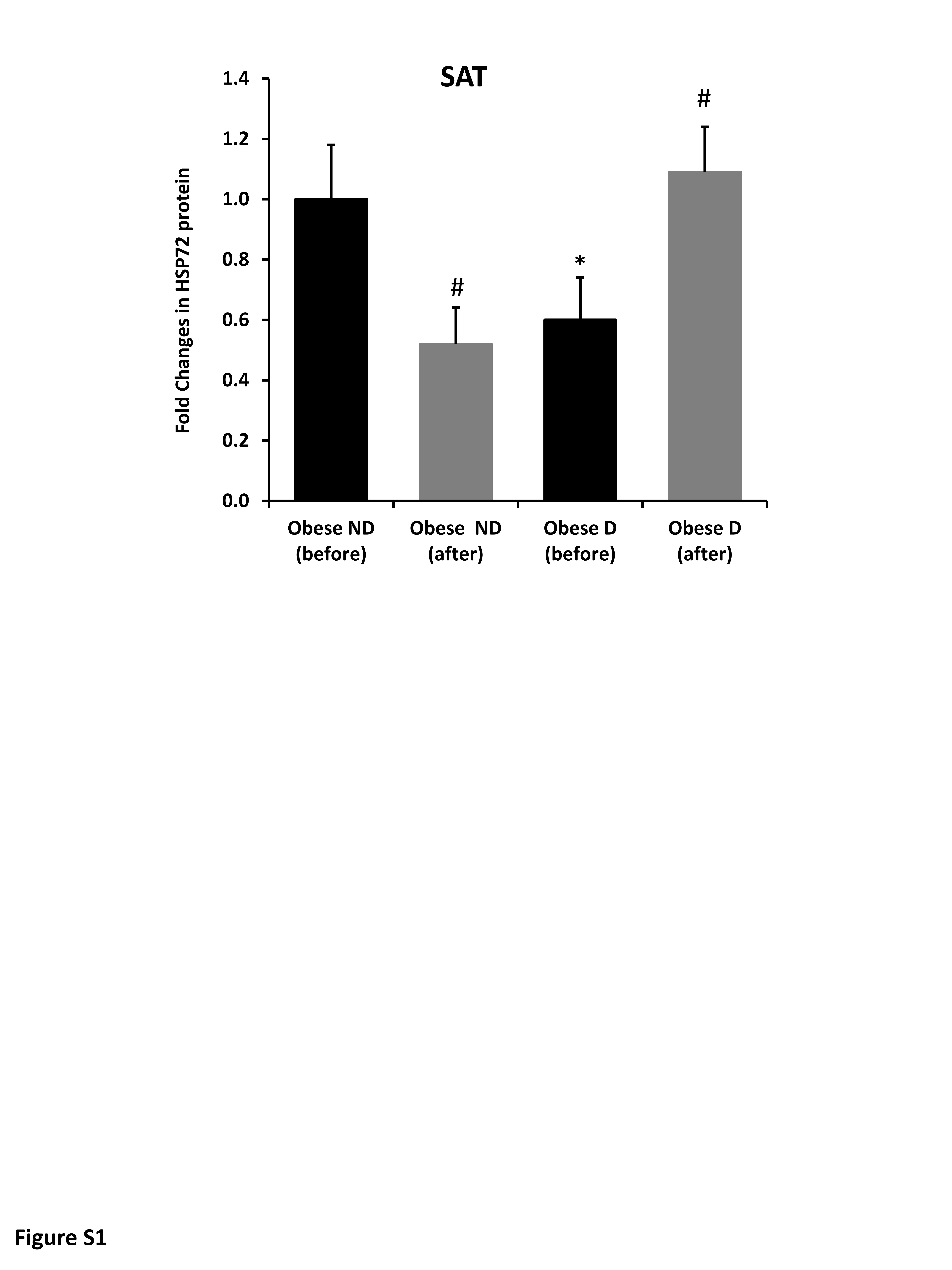

Supplement: Figure S1 — Decreased expression of HSP72 and its modulation by exercise in the subcutaneous adipose tissue (SAT) of obese subjects with diabetes. Immunohistochemical analysis of HSP72 expression in SAT sections from obese people without (ND) and with diabetes (D) before and after a 3-month physical exercise intervention (n = 10 for each group). Data are presented as fold changes in the diabetes group compared with that in the non-diabetes group. The p-value was determined using the Mann–Whitney test for comparisons between the diabetes and non-diabetes groups and using a paired t-test for intragroup comparisons before and after exercise. * denotes p < 0.05 between the diabetes and non-diabetes groups, and # denotes p < 0.05 between before and after exercise. [file image_1.jpeg]

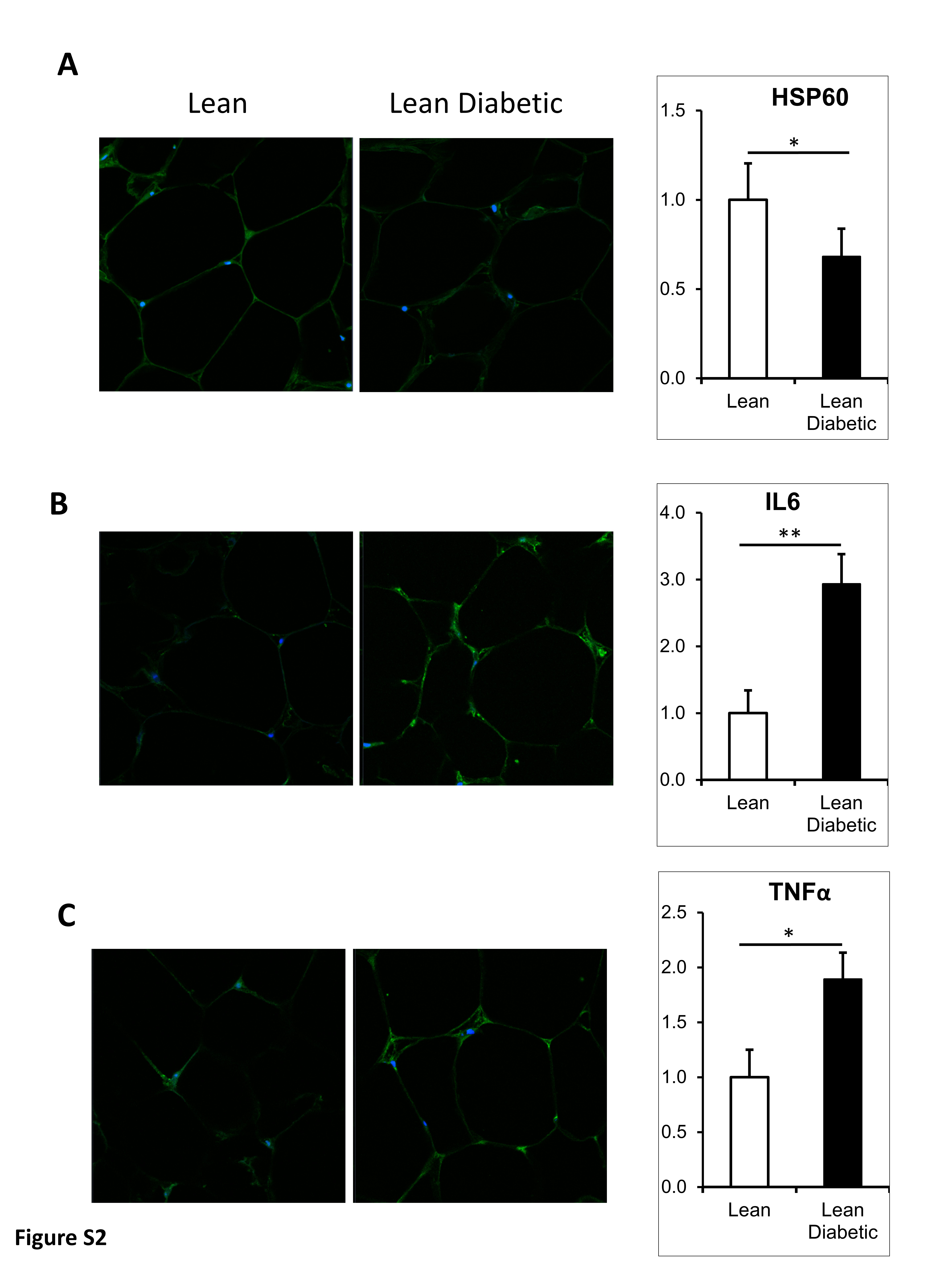

Supplement: Figure S2 — Expression of HSP60, IL-6, and TNF-α in the subcutaneous adipose tissue (SAT) of lean subjects with diabetes. Representative confocal immunofluorescence images illustrating HSP60 (A), IL-6 (B), and TNF-α (C) expression and localization in SAT from lean people with and without diabetes (n = 3 for each group). Quantification of the staining in SAT slides was performed as mentioned in Section “Materials and Methods.” [file image_2.jpeg]
