# Supplementary material for: Physical Exercise Enhanced Heat Shock Protein 60 Expression and Attenuated Inflammation in the Adipose Tissue of Human Diabetic Obese
Source: Front Endocrinol (Lausanne). 2018 Feb 6;9:16. doi: 10.3389/fendo.2018.00016 (PMC5808138; doi:10.3389/fendo.2018.00016)
Supplement: Supplementary file 3 [file table_1.docx]

**Supplementary table 1:** Primer sequences used for quantitative real time PCR to analyze Hsp60 and gapdh gene expression status.

| **Genes** | **Forward primers** | **Reverse primers** |
| --- | --- | --- |
| *Hsp-60* | 5’-GATGTCCTGGGCTGTTTCAT-3’ | 5’-GCCTCGATCAAACTTCATGC-3’ |
| *gapdh* | 5’-AGGGCTGCTTTTAACTCTGGT-3’ | 5’-CCCCACTTGATTTTGGAGGGA-3’ |
